# The socioeconomic dynamics of trends in female genital mutilation/cutting across Africa

**DOI:** 10.1136/bmjgh-2020-003088

**Published:** 2020-10-13

**Authors:** Ewa Batyra, Ernestina Coast, Ben Wilson, Valeria Cetorelli

**Affiliations:** 1 Population Studies Center, University of Pennsylvania, Philadelphia, Pennsylvania, USA; 2 Department of International Development, London School of Economics and Political Science, London, UK; 3 Department of Methodology, London School of Economics and Political Science, London, UK; 4 Department of Sociology, Stockholm University, Stockholm, Sweden; 5 Headquarters, United Nations Relief and Works Agency for Palestine Refugees in the Near East, Amman, Jordan

**Keywords:** child health, maternal health, public health

## Abstract

**Background:**

The majority of women who undergo female genital mutilation/cutting (FGM/C) live in Africa. Although the UN Sustainable Development Goals call for intensified efforts to accelerate the abandonment of FGM/C, little is known about where in Africa the declines in prevalence have been fastest and whether changes in prevalence differ by women’s socioeconomic status.

**Methods:**

We use data from Demographic and Health Surveys and Multiple Indicator Cluster Surveys for 23 African countries, collected between 2002 and 2016, and covering 293 170 women. We reconstruct long-term cohort trends in FGM/C prevalence spanning 35 years, for women born between 1965 and 1999. We compute absolute and relative changes in FGM/C prevalence and differentials in prevalence by women’s education and urban-rural residence. We examine whether socioeconomic differences in FGM/C are converging or diverging.

**Findings:**

FGM/C prevalence has declined fastest (in relative terms) in countries with lower initial prevalence, and more slowly in countries with higher initial prevalence. Although better-educated women and those living in urban areas tend to have lower prevalence, in some countries the opposite pattern is observed. Socioeconomic differentials in FGM/C have grown in the majority of countries, particularly in countries with moderate-to-higher overall prevalence.

**Conclusions:**

The documented relationship between absolute and relative FGM/C prevalence rates suggests that in settings with higher initial prevalence, FGM/C practice is likely to be more entrenched and to change more slowly. There is substantial variation between countries in socioeconomic differentials in prevalence and their changes over time. As countries change from higher to lower overall prevalence, socioeconomic inequalities in FGM/C are increasing.

**Key questions**

**What is already known?**

- Previous studies have documented trends in female genital mutilation/cutting (FGM/C) prevalence rates across African countries, but they are limited to analyses of population-level changes in prevalence and absolute changes in prevalence rates.
- These studies identified that in the majority of African countries, FGM/C prevalence has been decreasing over time.
- There are currently no studies examining either relative changes in FGM/C prevalence to identify the countries in which prevalence has changed fastest, or disaggregating trends in FGM/C prevalence rates by women’s socioeconomic characteristics, across African countries.

**What are the new findings?**

- Our analyses include the estimation of historic, absolute and relative changes in FGM/C prevalence rates as well as disaggregating trends by women’s education and urban-rural residence, for 23 African countries.
- We show that there is a relationship between absolute and relative FGM/C prevalence rates and that comparisons of trends across countries are dependent on the type of prevalence measure that is used.
- Our analyses suggests the presence of an underlying FGM/C transition and uncovers growing socioeconomic inequalities in FGM/C as countries change from high to low prevalence.

**What do the new findings imply?**

- Progress in the abandonment of FGM/C has not been uniform across socioeconomic groups.
- Interventions towards the abandonment of FGM/C must be designed to acknowledge and accommodate the socioeconomic heterogeneity in prevalence that we document.
- This study enhances understanding of the dynamics in FGM/C in Africa that is crucial for considering plausible future pathways and for developing theories of change in FGM/C prevalence.

## Introduction

More than 200 million women and girls alive today are estimated to have undergone female genital mutilation/cutting (FGM/C), most of whom live in Africa.^1^ In 2015, the United Nations General Assembly agreed a series of Sustainable Development Goals (SDGs), including a specific target to eliminate FGM/C by 2030.^2^ FGM/C is defined as ‘*all procedures involving partial or total removal of the external female genitalia or other injury to the female genital organs for non-medical reasons’* and is classified by severity into four categories.^3^ Despite sociocultural variations in FGM/C practices, it is widely recognised as a violation of human rights and harmful to the health of women and girls.^4^ The SDGs call for intensified national efforts to accelerate FGM/C elimination, but there is limited evidence to evaluate change across countries.^5^ Many studies have compared FGM/C prevalence in different African countries,^6–14^ but only a few studies have considered change in FGM/C prevalence rates over time.^15–20^ Little is known about the dynamics of change in FGM/C prevalence between and within African countries, in particular with respect to cohort dynamics (ie, changes over time in aggregate prevalence driven by behavioural change among younger generations).^21 22^

First, there is little evidence about the rate of change in practice of FGM/C, including research that identifies where declines in prevalence have been fastest. A few studies have compared long-run trends in adult prevalence rates for African countries where FGM/C is concentrated,^17 19 20^ but none of these have estimated both absolute and relative changes in prevalence.

Second, although cross-sectional studies have shown that FGM/C rates differ according to women’s socioeconomic status,^6–15 18^ there is little cross-national evidence about whether socioeconomic differentials in FGM/C are changing (where socioeconomic differentials are defined as a comparison between members of a similar cohort group who have different social or economic characteristics).^22^ Prevalence is declining for most types of FGM/C, including substitution of more severe with less severe types.^17^ For a limited number of countries, there is evidence that absolute declines in prevalence rates vary considerably by intracountry region, including the Central African Republic and Liberia.^15^ However, there is no systematic cross-country comparison of regional trends. Similarly, there is a lack of cross-national evidence to understand whether FGM/C prevalence is changing at different rates by socioeconomic factors.

These are important gaps in research. By analysing the dynamics of FGM/C prevalence, we can identify and begin to understand inequalities and—given evidence of declines—the extent to which subgroups are leading the transition towards low (or lower) prevalence. Accurate estimates of FGM/C dynamics are also vital if evidence-based policies are to be designed and evaluated,^23–25^ as well as in order to ensure the accuracy of projections of future FGM/C prevalence, including assessments of whether the SDG target is likely to be met.

This study compares and contrasts long-term trends in FGM/C prevalence across 23 African countries. It extends understanding by estimating absolute and relative long-term cohort trends in FGM/C prevalence—by education and urban-rural residence—using harmonised data that are comparable across all 23 countries. We use a completed cohort approach based on estimation of nationally-representative prevalence rates for birth cohorts of adults who are no longer at risk of FGM/C, over 35 years. This approach offers more stable estimates of prevalence by excluding those who may be at future risk of FGM/C and greater insight into underlying socioeconomic dynamics of behaviours and experiences that are more comparable across cohorts. Prior comparative studies of FGM/C prevalence trends using a cohort perspective^15 17 19 20^ neither consider relative change nor urban-rural or educational dynamics.

Our approach allows us to demonstrate how the dynamics of FGM/C in Africa are changing. We identify countries where FGM/C prevalence is static, rising or falling, as well as where it has been changing fastest in relative and absolute terms. By exploring the relationship between absolute and relative changes in FGM/C prevalence rates, we link our empirical findings to theories that aim to explain FGM/C decline (or lack thereof), specifically the Theory of Social Convention.^26^ By including the degree of socioeconomic heterogeneity in FGM/C dynamics, we additionally show which groups are leading the decline in FGM/C and provide much-needed evidence for evaluating future changes in FGM/C prevalence.

## Methods

Our methods include three stages: (1) data harmonisation, (2) estimating trends in absolute and relative FGM/C prevalence for each country by birth cohort and (3) analysing educational and urban-rural differentials in these trends.

### Data harmonisation

For each country, we either use data from the Demographic and Health Survey (DHS) or the Multiple Indicator Cluster Survey (MICS) (table 1). We focus on countries that had at least one survey including an FGM/C module and where FGM/C practice is non-negligible (national prevalence of at least 5%). Three countries (Cameroon, Niger and Uganda) were excluded from the analysis due to a very small number of women undergoing FGM/C reflecting national prevalence of 1%–2%.

**Table 1:** Countries, data sources and sample sizes

	Country abbr.	Survey type	Survey year	Oldest cohort	Youngest cohort	Sample size
Benin	BJ	DHS	2011/12	1965–69	1995–97	16 152
Burkina Faso	BF	DHS	2010	1965–69	1990–94	15 430
Central African Republic	CF	MICS	2010	1965–69	1990–94	10 562
Chad	TD	DHS	2014/15	1965–69	1995–99	11 402
Côte d’Ivoire	CI	DHS	2011/12	1965–69	1995–97	9708
Djibouti	DJ	MICS	2006	1965–69	1990–91	5471
Egypt	EG	DHS	2014	1965–69	1995–99	21 441
Eritrea	ER	DHS	2002	1965–69	1985–87	6659
Ethiopia	ET	DHS	2005	1965–69	1985–89	11 367
Gambia	GM	DHS	2013	1965–69	1995–98	10 060
Ghana	GH	MICS	2011	1965–69	1995–96	9992
Guinea	GN	DHS	2012	1965–69	1995–97	8852
Guinea-Bissau	GW	MICS	2014	1965–69	1995–99	10 193
Kenya	KE	DHS	2014	1965–69	1995–99	14 682
Mali	ML	DHS	2012/13	1965–69	1995–97	10 259
Mauritania	MR	MICS	2015	1965–69	1995–99	13 612
Nigeria	NG	DHS	2013	1965–69	1995–98	35 983
Senegal	SN	DHS	2014	1965–69	1995–99	8453
Sierra Leone	SL	DHS	2013	1965–69	1995–98	16 371
Somalia	SO	MICS	2006	1965–69	1990–91	6241
Sudan	SD	MICS	2014	1965–69	1995–99	18 292
Tanzania	TZ	DHS	2015/16	1965–69	1995–99	12 619
Togo	TG	DHS	2013/14	1965–69	1995–99	9369
*Total*						293 170

DHS, Demographic and Health Survey; MICS, Multiple Indicator Cluster Survey.

Both DHS and MICS are representative of national populations, have similar designs and are comparable. Our direct estimation of FGM/C prevalence relies on the quality of these data. We note that, although prior research has highlighted issues relating to data quality, such as the potential for some DHS survey questions to suffer from recall and reporting bias,^27^ or interviewer effects,^28^ prior research also suggests that these surveys are of sufficient quality for estimating FGM/C prevalence (as long as they avoid disaggregating by the type of practice, which is not something we do here).^29^ Moreover, the quality of these data are deemed sufficient in order to form the basis of indirect estimates of FGM/C prevalence among immigrants in high-income countries.^30–36^ We harmonised all 23 national surveys to ensure that questions and response codes are similar, and that the data are of sufficient quality for analysis. We use the same approach for each country, thereby facilitating a direct comparison within and between countries over time. During this harmonisation, we corresponded with DHS/MICS support teams and completed quality assurance checks of our derived variables, including those for age and birth cohort, which are essential for the validity of our time series estimates. This quality assurance resulted in minimal changes to the data, and essentially involved renaming variables and recoding the values of variables (so that codes were consistent across surveys including for missing values). Our analyses account for the design of each survey using appropriate survey weights and the ‘SVY’ command in Stata V.14.^37^

### Estimating trends in absolute and relative FGM/C prevalence for each country and by birth cohort

We restrict our analysis to women aged 15–49 years in order to generate comparable time-series estimates of completed FGM/C prevalence rates. Of those who undergo FGM/C, in all countries in our study, almost all women did so before age 15. Those under 15 at the time of each survey are judged to be still at risk of FGM/C and are excluded from analysis. All the FGM/C modules include a question: “Have you yourself ever been circumcised?” which we use to calculate prevalence, that is, the percentage of women in a given cohort who report having undergone FGM/C. The potential limitation of such self-reported data is that women may be unwilling to disclose having undergone FGM/C due to the sensitivity of the subject or its illegality in some countries,^15^ or they may be unaware that they have been cut, especially if that happened at a young age.^38^ Despite these limitations, such self-reported data are the only source of information of FGM/C that can be used for comprehensive comparative analyses. Table S1, online supplemental material shows that missing information about women’s FGM/C status is below 5% in all countries except for Chad (35%) and Kenya (53%). There are no substantial differences in the level if missing values by education level or place of residence in Chad and Kenya. We retain these two countries in our analysis, but the results should be interpreted while keeping in mind high levels of missing information on FGM/C status.

10.1136/bmjgh-2020-003088.supp1 Supplementary data

We reconstruct historic trends in prevalence using data on year of birth, grouped into 5-year birth cohorts, using one cross-sectional survey for each country. The range of cohorts that can be included depends on the date of each national survey. Since the majority of countries conducted their latest survey around 2010, we use surveys conducted just before or after 2010, even if a more recent survey is available. This is in order to generate trends that are comparable between countries. For most countries, we are able to estimate FGM/C prevalence for women born between 1965–69 and 1995–99. However, the upper end of this range is more restricted for some countries, most notably Burkina Faso, Central African Republic, Djibouti, Eritrea, Ethiopia and Somalia (table 1). We conduct additional analysis using 3-year, instead of 5-year, birth cohorts to make sure that our results are not sensitive to alternative ways of grouping cohorts. We show these rates in the online supplemental material, table S2.

We then calculate an additional time series (for each country) that enables a cross-national comparison of relative change in FMG/C over time. This is done by indexing aggregate prevalence rates for each cohort to the national prevalence for women born 1965–69 and allows us to examine similarities and differences in the rate of change in FGM/C prevalence between countries.

### Analysing educational and urban-rural differentials

Factors influencing the practice of FGM/C are dynamic, heterogeneous and context-specific.^15^ To explore the determinants of FGM/C, drivers of change and dynamics of inequality, we focus on several indicators of socioeconomic status. These are chosen from the indicators that were available and equivalent (ie, comparable) in all surveys. After examining all possible indicators, only two—education and place of residence—were sufficiently comparable to warrant inclusion. These two factors are known to be associated with FGM/C practices,^6–14 18^ but are not the only factors. A third factor—household wealth—was identified for potential inclusion. However, it is well-known that wealth is more difficult to standardise and compare than other socioeconomic measures, in part because of its conceptual complexity.^39–41^ It is for this reason that we analyse wealth, but restrict its presentation to the supplementary material due to concerns about validity and the fact that wealth index information is not available for all surveys (see online supplemental figure S1). We considered but were unable to calculate consistent and comparable trends by other factors including ethnicity and religion, in part due to the unavailability of questions for all countries, and in part due to the difficulty of cross-national harmonisation of these variables (and their categories).

We calculate the difference in absolute prevalence for: (a) women who have no education vs those who have some education and (b) women who live in rural areas versus those who live in urban areas. Having some education is defined as having completed any years of schooling. We use the classification of urban-rural areas as defined in a given survey for each country, which is based on each country’s urban-rural definition at the time of the survey,^42^ and is one of the most common ways of operationalising this concept for the analysis of socioeconomic dynamics and development.^22 43^ Both education and residence variables are recorded at the time of the survey, and do not capture an individual’s characteristics at the time of FGM/C. While this fact has no implications for the analysis of educational differences, it is a potential limitation for the study of urban-rural differences because migration between rural and urban areas may differ by FGM/C status. We are not aware of evidence that indicates whether this is the case, but we acknowledge this limitation and later discuss its possible influence on our results. The socioeconomic composition of the population (the per cent of women with some education and living in urban areas) is presented in online supplemental material, table S3.

### Patient and public involvement

This research was carried out without any public involvement, essentially because it was not feasible to involve members of the public given the time and funding that were available.

## Results

**Figure 1:**
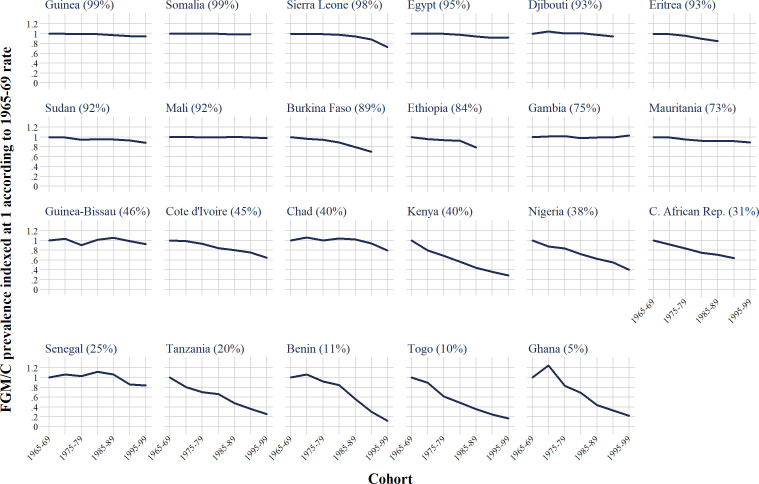
Indexed time series of trends in female genital mutilation/cutting (FGM/C) prevalence by birth cohort (relative change in FGM/C prevalence). Countries are sorted according to the national FGM/C prevalence rate for 1965-69 (oldest) cohort, which is shown in the parentheses.

In general, there has been a decline in the prevalence of FGM/C, however there is stark variation across countries in long-run trends (figure 1). For women born in 1965–69, prevalence varies from 5% in Ghana (the lowest in our study) to 99% in Guinea (the highest). In some countries, such as Somalia and Mali, there has been no decline in prevalence, and in Gambia there appears to have been a small increase. In Sierra Leone, the rate has fallen by 27% for women born 30 years later, reflected in a change in the indexed FGM/C prevalence from 1 to 0·73 between 1965–69 and 1995–99. There is no obvious pattern by region, for example, the neighbouring countries of Gambia, Senegal and Mauritania all exhibit very different trends. Gambia is the only country in our study where FGM/C prevalence increased over the 30-year period; prevalence fell steadily in Mauritania by around 2%–3% per year, whereas prevalence in Senegal increased for cohorts born 1970–89, but declined rapidly thereafter.

Alongside Sierra Leone, several other countries in the top two quartiles of prevalence (top two rows of figure 1) exhibit material declines in prevalence of >10%: Sudan (11%), Eritrea (15%), Ethiopia (21%) and Burkina Faso (30%). Nonetheless, the pace of decline in these countries is generally slower as compared with countries of below average prevalence (bottom two rows of figure 1). Most of these lower prevalence countries have seen a reduction of >30% in FGM/C prevalence across three decades, with the exception of Chad, Senegal and Guinea-Bissau (reductions of 20%, 16% and 8%, respectively). Different countries show the ‘largest’ reduction, depending on whether the reduction is calculated in absolute or relative terms. When comparing those born 1965–69 with the most recent cohorts, the largest absolute declines are for Kenya (28 percentage points), Burkina Faso (27), Sierra Leone (27), Nigeria (23) and Ethiopia (18) (absolute FGM/C prevalence rates for the total population are shown in online supplemental table S4). These are countries with substantial relative declines as well (71%, 30%, 27%, 60% and 21%, respectively). Nonetheless, the largest relative declines over the same period occurred in Tanzania, Benin, Togo and Ghana (declines of 74%, 88%, 83% and 78%, respectively).

**Figure 2:**
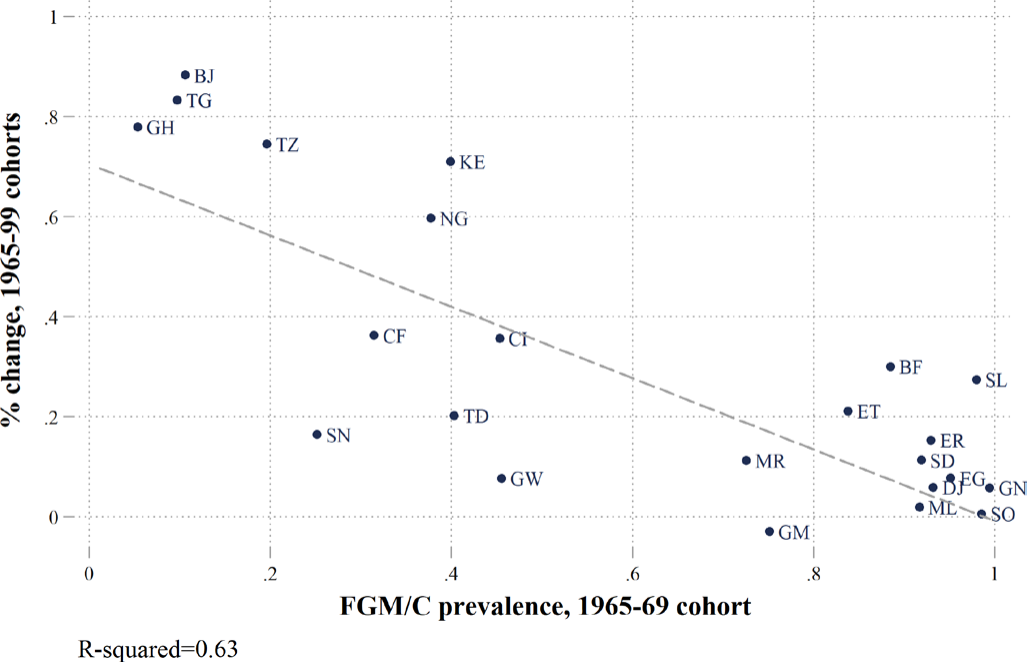
Relationship between initial female genital mutilation/cutting (FGM/C) prevalence (1965–69 cohort) and the rate of change. Note: the percentage change is the change in FGM/C prevalence rates between those born 1965–69 (oldest cohort) and the youngest cohort available for each country.

Another pattern that emerges from our analysis of long-term trends is the relationship between ‘initial’ absolute FGM/C prevalence, for those born 1965–69 (index=100), and the relative change in prevalence for subsequent cohorts (R^2^=0·63). Countries with the highest prevalence for those born in 1965–69 also exhibit a much smaller relative change in prevalence for cohorts born over the next three decades (figure 2). The largest relative declines are for countries where FGM/C was already a minority practice for the 1965–69 birth cohort.

**Figure 3:**
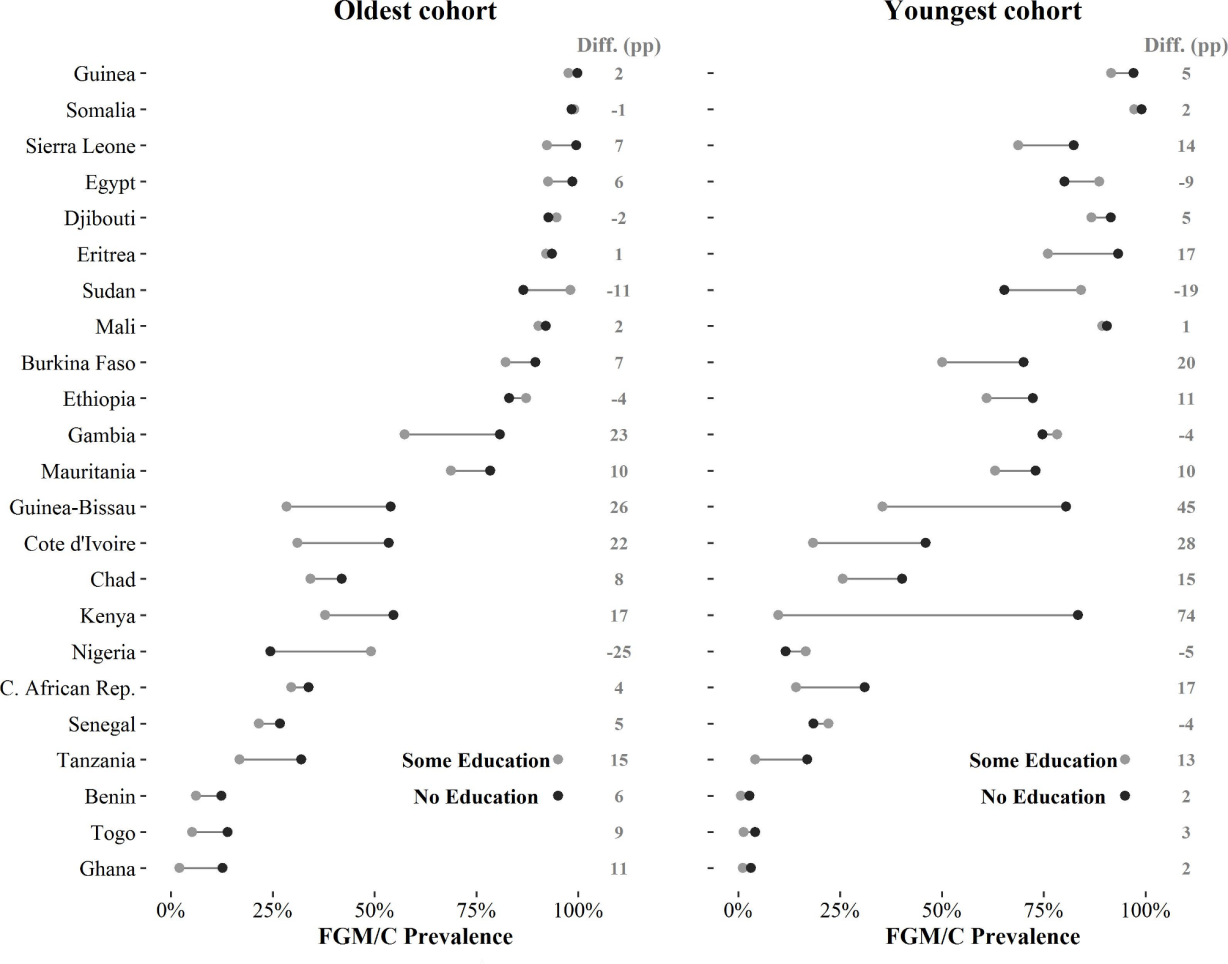
Absolute FGM/C prevalence rates by education level, percentage point (pp) difference (diff.) in female genital mutilation/cutting (FGM/C) prevalence between women with no education and some education (diff. (pp)), oldest and youngest cohorts. Note: countries are ranked according to the national FGM/C prevalence rates of the 1965–69 cohorts (oldest cohort). The youngest cohorts are listed in table 1.

**Figure 4:**
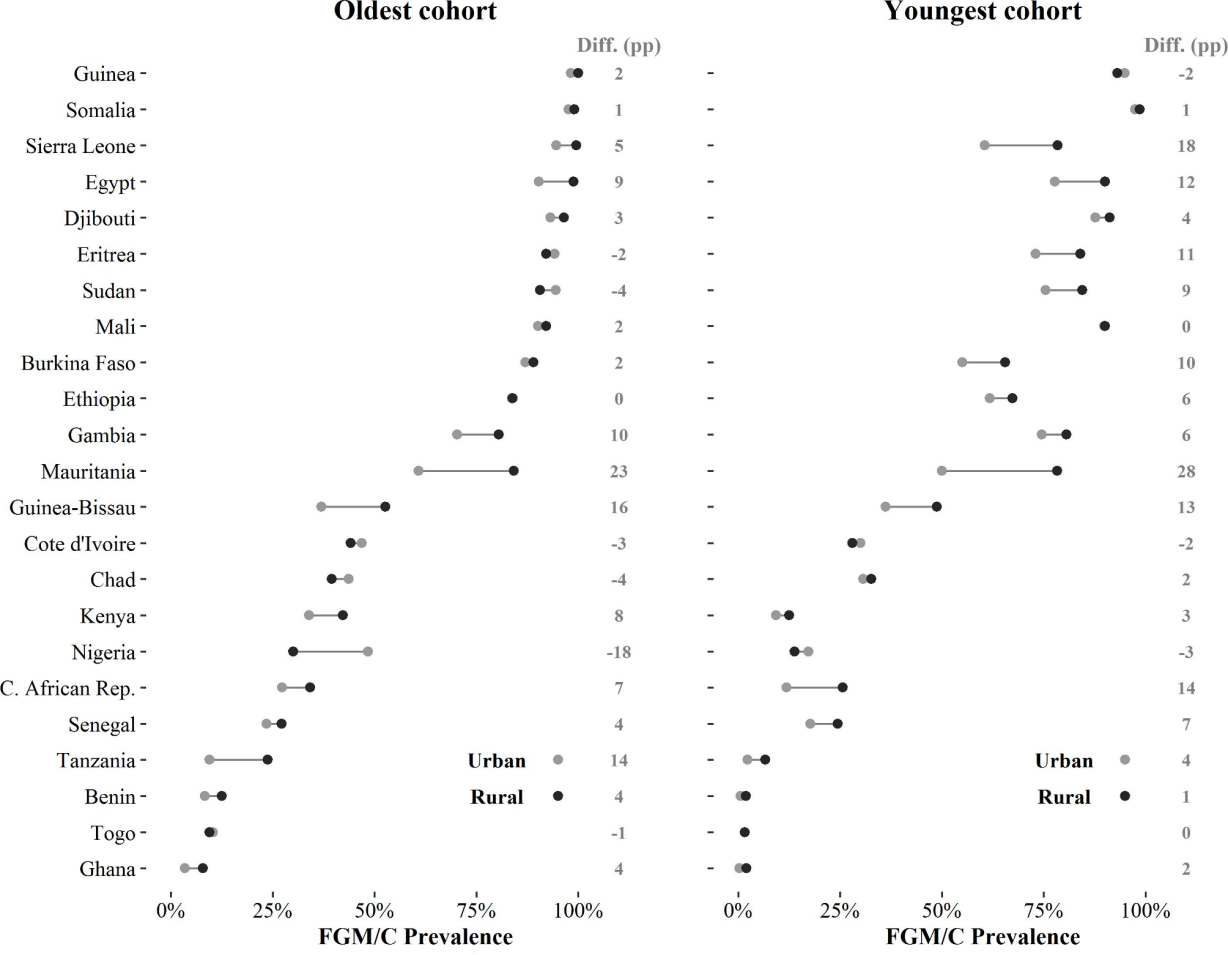
Absolute female genital mutilation/cutting (FGM/C) prevalence rates by place of residence, percentage point (pp) difference (diff.) in FGM/C prevalence between women in rural areas and urban areas (diff. (pp)), oldest and youngest cohorts. Note: countries are ranked according to the national FGM/C prevalence rates of the 1965–69 cohorts (oldest cohort). The youngest cohorts are listed in table 1.

There is considerable cross-national variation in the magnitude of educational and urban-rural differences in FGM/C prevalence. Figures 3 and 4 show absolute FGM/C prevalence rates by socioeconomic characteristics; countries are ranked by the aggregate FGM/C prevalence rate of the 1965-69 cohorts (relative FGM/C prevalence rates by education level and place of residence are shown in the online supplemental table S4). For women born 1965–69, those with no education have a much higher prevalence in Guinea-Bissau, Gambia, Côte d’Ivoire and Kenya (differentials of >15 percentage points as compared with those who have any education) (figure 3). In some countries (eg, Sudan, Nigeria), women with some education from these cohorts have a higher prevalence than women with no education, and the difference is >10 percentage points. This shows that education is not a good predictor of FGM/C cross-nationally, although it is notable that educational differentials among women born in 1965–69 are largest in countries with moderate levels of overall prevalence, and smallest in countries with the highest prevalence.

The heterogeneity that is evident for women born in 1965–69 is amplified when examining how differentials have changed over time. The trends in differentials move in different directions across countries, including in divergent ways for educational differentials, and there is also sizeable variation in the changing magnitude of differentials. For example, in Central African Republic, the difference in prevalence between women with no education and some education increases from 4 percentage points for the 1965–69 cohort to 17 percentage points for the 1990–94 cohort. By contrast, in Côte d’Ivoire where there is also a sizeable differential by education, it is much more stable over time. In Ghana, the same educational differential changes in the opposite direction, decreasing from 11 to 2 percentage points.

These socioeconomic differentials can be interpreted as a measure of inequality in FGM/C practices. To this extent, countries can be categorised as becoming more or less equal if differentials become smaller or larger (in absolute terms). Countries trending towards equality include Gambia, Nigeria, Benin, Togo and Ghana. However, these countries appear to be in the minority as compared with those where inequality is widening, such as Sierra Leone, Burkina Faso, Eritrea, Ethiopia, Guinea-Bissau, Kenya or Central African Republic. Increases in educational differentials occurred in countries that had moderate-to-high levels of initial overall prevalence (ie, among the 1965–69 cohort). Consequently, for more recent cohorts, educational differentials are largest in countries with moderate-to-high prevalence. It is only among countries with the lowest initial prevalence rates—such as Benin, Togo and Ghana—that educational differentials have consistently reduced.

The variation in magnitude (and direction) of urban-rural differentials is less pronounced than for education (figure 4). In the majority of countries, women who lived in urban areas at the time of the survey have lower FGM/C prevalence than rural women, although in many cases the difference is small or negligible. For the oldest cohort, there is a much lower urban prevalence (>10 percentage points) in Guinea-Bissau, Mauritania and Tanzania. However, the opposite is true in Nigeria, Chad, Sudan, Côte d’Ivoire and Eritrea, where urban prevalence rates are higher (with differentials ranging from 2 to 18 percentage points). As with education, urban-rural differentials tend to be largest in countries with moderate levels of overall prevalence. For the latest cohorts, differentials remain largest in countries with moderate prevalence, and show an increase in countries with higher overall levels of prevalence (Sierra Leone, Egypt, Eritrea, Sudan and Burkina Faso). In drawing these conclusions, it is important to note that place of residence is recorded at the time of interview, such that urban-rural differentials also reflect patterns of (rural to urban) migration over time. Nevertheless, we frequently observe that differentials between rural and urban areas are increasing.

## Discussion

While FGM/C practices vary substantially across and within countries, a comparison of national trends can illuminate where, to what extent, and how rapidly change is occurring. Our results focus on the dynamics and heterogeneity of FGM/C within and between countries, and show the extent to which the practice is changing across Africa.

The analysis of relative changes in prevalence rates provides a novel perspective for looking at the evolution of FGM/C practices and allows us to identify where it has been changing fastest. Despite considerable variation across countries, we uncover a clear relationship between absolute rates and relative changes in FGM/C prevalence. Relative declines in cohort-specific prevalence rates have been faster in countries that began with low absolute levels of prevalence. These findings provide macro-level evidence in support of the Theory of Social Convention, as applied to FGM/C.^26^ In countries with lower initial prevalence rates, the mechanisms of social convention—such as marriageability or peer conventions that support the practice of FGM/C—are less prevalent and less likely to be reinforced because a minority of the population practices FGM/C.^44^ In settings with higher initial prevalence, these social conventions are more likely to be entrenched and less likely to change.

These macro-level patterns are not deterministic: high prevalence does not mean that high prevalence will persist. With the exception of a few countries such as Somalia, Mali and Gambia, even in the highest prevalence countries FGM/C prevalence has started to decline, although at a slow pace. However, we also document that, as in the cases of Senegal and Guinea-Bissau, prevalence is not necessarily decreasing monotonically: initial declines in prevalence do not always lead to subsequent declines.

We show substantial variation between countries in their socioeconomic differentials in FGM/C prevalence. Theories of diffusion suggest that certain socioeconomic groups—such as those with higher education—are more likely to adopt new (non-traditional) behaviours.^45^ Yet, we show that FGM/C differentials are not in the same direction across countries, and women with some education (or living in urban areas) can have higher average prevalence rates. As noted elsewhere, it is important to realise that different communities practice FGM/C in different social contexts, and that each context presents specific challenges for reformers.^46^ Interventions towards abandonment of FGM/C must be designed to acknowledge and accommodate the heterogeneity we document, not least with respect to generalisability.

Finally, we show how cross-national variation in socioeconomic differentials change over time. In the past, differentials were smallest in countries where the majority of the population practice FGM/C, and were largest in countries with moderate prevalence levels. However, socioeconomic inequalities in FGM/C have decreased in the lowest prevalence countries and increased in countries with moderate-to-high levels of prevalence. We speculate that these changes are, at least in part, driven by an underlying FGM/C transition, as countries change from high to low prevalence—or from FGM/C being a majority to a minority practice. Once a minority of the population practices FGM/C, then it appears that socioeconomic differences in the practice (begin to) disappear, at least at the national-level. As well as adding knowledge about the dynamics of change in FGM/C, these findings can inform research to develop and enhance theories of change, as well as in order to project future levels of FGM/C prevalence.

We recommend that future research seeks to develop an even richer understanding of socioeconomic trends in FGM/C—and the complex behaviour that determines these trends—using both qualitative and qualitative methods. Nevertheless, we hope that our evidence will help to enable programme directors, policy-makers, researchers and members of civil society to better understand changes in FGM/C that are crucial for considering plausible future policies.
