# Supplementary material for: The socioeconomic dynamics of trends in female genital mutilation/cutting across Africa
Source: BMJ Glob Health. 2020 Oct 13;5(10):e003088. doi: 10.1136/bmjgh-2020-003088 (PMC7554470; doi:10.1136/bmjgh-2020-003088)
Supplement: Supplementary data [file bmjgh-2020-003088supp001.pdf]

Supplementary material

Table S1: Per cent missing values for information about women's FGM/C status, by country, for total population and according to women's socioeconomic characteristics (%)

	total population	some education	no education	urban	rural
Benin	0.0	0.0	0.0	0.0	0.0
Burkina Faso	0.1	0.0	0.1	0.0	0.1
Central African Republic	0.2	0.3	0.2	0.3	0.2
Chad	35.1	34.6	35.4	36.5	34.7
Cote d'Ivoire	0.1	0.1	0.1	0.1	0.1
Djibouti	0.3	0.1	0.4	0.3	0.4
Egypt	0.0	0.0	0.0	0.1	0.0
Eritrea	0.0	0.1	0.0	0.1	0.0
Ethiopia	3.1	3.9	2.5	5.1	2.2
Gambia	0.3	0.3	0.2	0.3	0.2
Ghana	0.0	0.0	0.0	0.0	0.0
Guinea	0.1	0.1	0.1	0.2	0.0
Guinea-Bissau	0.0	0.0	0.0	0.0	0.0
Kenya	52.6	52.6	52.7	52.9	52.4
Mali	0.0	0.0	0.0	0.0	0.0
Mauritania	1.1	1.3	0.7	1.2	1.1
Nigeria	4.5	3.4	6.5	4.9	4.2
Senegal	0.0	0.0	0.0	0.0	0.0
Sierra Leone	0.1	0.1	0.1	0.1	0.1
Somalia	0.1	0.2	0.1	0.3	0.1
Sudan	0.1	0.1	0.1	0.0	0.1
Tanzania	0.0	0.0	0.0	0.0	0.0
Togo	0.1	0.1	0.1	0.1	0.1

Table S2: FGM/C prevalence by 3-year cohorts, total population (T), by education level (SE, NE) and place of residence (U, R), absolute rates (A) for the oldest (O) and the youngest (Y) cohorts, relative rates (R) for the youngest cohort

	Oldest cohort (O)	Youngest cohort (Y)	T-O-A	T-Y-A	T-Y-R	SE-O-A	SE-Y-A	SE-Y-R	NE-O-A	NE-Y-A	NE-Y-R	U-O-A	U-Y-A	U-Y-R	R-O-A	R-Y-A	R-Y-R
Guinea	1965-67	1995-97	1.00	0.94	0.94	0.98	0.92	0.93	1.00	0.97	0.97	0.98	0.95	0.96	1.00	0.93	0.93
Somalia	1965-67	1989-91	0.98	0.97	0.99	0.98	0.95	0.97	0.98	0.98	1.01	0.96	0.96	0.99	0.99	0.97	0.98
Sierra Leone	1965-67	1995-97	0.98	0.74	0.75	0.91	0.71	0.78	0.99	0.84	0.85	0.93	0.63	0.68	0.99	0.81	0.82
Egypt	1965-67	1995-97	0.95	0.89	0.93	0.93	0.90	0.97	0.98	0.80	0.82	0.91	0.79	0.87	0.99	0.91	0.93
Sudan	1965-67	1995-97	0.94	0.82	0.88	0.98	0.85	0.87	0.89	0.66	0.74	0.95	0.77	0.81	0.93	0.85	0.92
Eritrea	1965-67	1983-85	0.93	0.79	0.84	0.91	0.77	0.84	0.95	0.86	0.90	0.92	0.74	0.80	0.94	0.82	0.87
Djibouti	1965-67	1989-91	0.93	0.89	0.95	0.95	0.88	0.92	0.92	0.91	0.99	0.93	0.89	0.96	0.96	0.90	0.94
Mali	1965-67	1995-97	0.92	0.90	0.98	0.91	0.89	0.98	0.92	0.91	0.99	0.90	0.90	0.99	0.92	0.90	0.98
Burkina Faso	1965-67	1992-94	0.88	0.58	0.66	0.82	0.47	0.57	0.89	0.67	0.76	0.87	0.52	0.60	0.88	0.61	0.69
Ethiopia	1965-67	1986-88	0.83	0.65	0.78	0.87	0.62	0.71	0.83	0.70	0.84	0.87	0.61	0.70	0.83	0.67	0.81
Gambia	1965-67	1995-97	0.74	0.77	1.05	0.52	0.78	1.51	0.80	0.74	0.92	0.68	0.74	1.08	0.79	0.81	1.02
Mauritania	1965-67	1995-97	0.74	0.64	0.86	0.68	0.62	0.92	0.80	0.70	0.87	0.60	0.49	0.82	0.87	0.78	0.90
Cote d'Ivoire	1965-67	1995-97	0.46	0.29	0.64	0.37	0.18	0.49	0.50	0.46	0.92	0.52	0.30	0.58	0.40	0.28	0.70
Guinea-Bissau	1965-67	1995-97	0.42	0.43	1.01	0.28	0.36	1.29	0.49	0.80	1.63	0.33	0.37	1.14	0.50	0.50	1.00
Kenya	1965-67	1995-97	0.40	0.12	0.29	0.38	0.10	0.25	0.49	0.89	1.81	0.31	0.09	0.30	0.43	0.13	0.29
Nigeria	1965-67	1995-97	0.37	0.16	0.42	0.48	0.17	0.35	0.26	0.12	0.47	0.48	0.17	0.35	0.30	0.14	0.49
Chad	1965-67	1995-97	0.35	0.33	0.94	0.28	0.25	0.90	0.37	0.43	1.14	0.36	0.32	0.89	0.35	0.34	0.96
C.A. Republic	1965-67	1992-94	0.33	0.18	0.54	0.31	0.13	0.41	0.35	0.29	0.82	0.26	0.10	0.40	0.37	0.23	0.61
Senegal	1965-67	1995-97	0.24	0.21	0.88	0.20	0.22	1.11	0.26	0.19	0.73	0.23	0.18	0.80	0.27	0.25	0.92
Tanzania	1965-67	1995-97	0.19	0.05	0.29	0.14	0.04	0.31	0.34	0.17	0.51	0.05	0.02	0.33	0.24	0.08	0.32
Benin	1965-67	1995-97	0.12	0.01	0.10	0.05	0.01	0.14	0.15	0.03	0.19	0.09	0.01	0.06	0.14	0.02	0.13
Togo	1965-67	1995-97	0.10	0.02	0.21	0.06	0.02	0.31	0.13	0.05	0.34	0.10	0.02	0.20	0.09	0.02	0.22
Ghana	1965-67	1992-94	0.05	0.02	0.37	0.02	0.01	0.63	0.12	0.12	1.04	0.02	0.01	0.59	0.08	0.02	0.28

Note: O-oldest cohort, Y-youngest cohort, T-total population, SE-some education, NE-no education, U-urban, R-rural, A-absolute rate, R-relative rate. Countries are ranked according to the national FGM/C prevalence rates of the 1965-67 cohorts. Relative rates for the oldest cohort are equal to 1.

Figure S1: Absolute FGM/C prevalence rates by household wealth quantile, percentage point (pp) difference (Diff.) in female genital mutilation/cutting (FGM/C) prevalence between women living in the richest and richer households (top 40% - Rich) and women living in the poorest and poorer households (bottom 40% - Poor) (Diff. (pp)), oldest and youngest cohorts

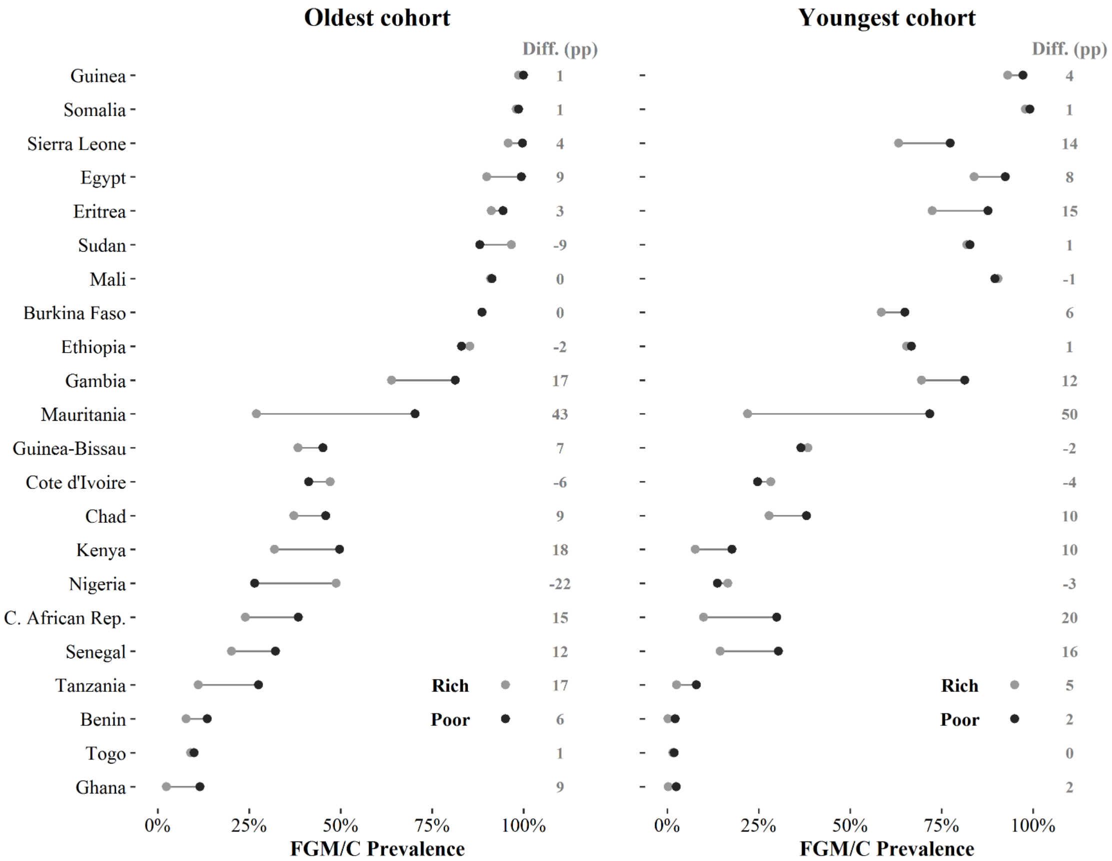

Note: Countries are ranked according to the national FGM/C prevalence rates of the 1965-69 cohorts (oldest cohort). The youngest cohorts as listed in table 1. Wealth index is expressed in terms of quintiles that divide households according to wealth: (1) poorest, (2) poorer, (3) middle, (4) richer and (5) richest. Wealth index is not available for Djibouti 2006 MICS and this country is omitted from the figure.

Table S3: Per cent of women with some education and living in urban areas, oldest and youngest cohorts (%)

	% Some Education		% Urban	
**Country /Cohort**	**Oldest**	**Youngest**	**Oldest**	**Youngest**
Benin	28.8	73.9	45.5	49.1
Burkina Faso	13.5	48.5	22.5	25.2
Central African Republic	54.6	65.3	39.1	40.7
Chad	20.8	55.2	20.3	26.4
Cote d'Ivoire	36.3	60.7	45.6	59.3
Djibouti	29.2	77.2	96.8	97.5
Egypt	55.9	90.6	42.4	17.7
Eritrea	34.9	84.1	43.4	47.0
Ethiopia	19.6	54.4	14.0	21.3
Gambia	23.8	76.0	51.7	52.6
Ghana	69.4	97.2	55.8	48.6
Guinea	14.3	60.0	27.8	40.3
Guinea-Bissau	32.8	85.3	45.0	53.1
Kenya	87.7	97.6	29.8	30.3
Mali	13.7	48.9	19.1	31.9
Mauritania	60.1	86.4	49.7	49.0
Nigeria	52.8	71.7	41.9	41.5
Senegal	31.2	69.9	54.8	50.0
Sierra Leone	20.1	81.7	30.4	40.3
Somalia	31.2	57.0	32.2	45.3
Sudan	46.9	85.9	34.9	32.5
Tanzania	81.4	93.5	28.7	37.9
Togo	47.6	89.4	35.1	44.5

Note: The oldest and youngest cohorts as listed in table 1.

Table S4: FGM/C prevalence for total population for the oldest and youngest cohorts (absolute rates) and FGM/C prevalence for the youngest cohort indexed at 1 according to 1965-69 rate (relative rates) by education level and place of residence.

	total population, oldest cohort (absolute rate)	total population, youngest cohort (absolute rate)	some education, youngest cohort, (relative rate)	no education, youngest cohort, (relative rate)	urban, youngest cohort, (relative rate)	rural, youngest cohort, (relative rate)
Guinea	0.99	0.94	0.94	0.97	0.97	0.93
Somalia	0.99	0.98	0.98	1.01	1.00	0.99
Sierra Leone	0.98	0.71	0.74	0.83	0.64	0.79
Egypt	0.95	0.88	0.96	0.81	0.86	0.91
Djibouti	0.93	0.88	0.92	0.99	0.94	0.95
Eritrea	0.93	0.79	0.83	1.00	0.77	0.91
Sudan	0.92	0.82	0.86	0.75	0.80	0.93
Mali	0.92	0.90	0.99	0.98	1.00	0.98
Burkina Faso	0.89	0.62	0.61	0.78	0.63	0.74
Ethiopia	0.84	0.66	0.70	0.87	0.74	0.80
Gambia	0.75	0.77	1.37	0.93	1.06	1.00
Mauritania	0.73	0.64	0.92	0.93	0.82	0.93
Guinea-Bissau	0.46	0.42	1.25	1.49	0.98	0.93
Cote d'Ivoire	0.45	0.29	0.59	0.86	0.64	0.64
Chad	0.40	0.32	0.75	0.96	0.70	0.83
Kenya	0.40	0.12	0.26	1.53	0.27	0.30
Nigeria	0.38	0.15	0.34	0.48	0.36	0.46
C.A. Republic	0.31	0.20	0.48	0.92	0.43	0.75
Senegal	0.25	0.21	1.03	0.69	0.75	0.90
Tanzania	0.20	0.05	0.25	0.53	0.24	0.28
Benin	0.11	0.01	0.11	0.23	0.07	0.15
Togo	0.10	0.02	0.26	0.30	0.16	0.18
Ghana	0.05	0.01	0.53	0.25	0.09	0.26

Note: Countries are ranked according to the national FGM/C prevalence rates of the 1965-69 cohorts. The oldest and youngest cohorts as listed in table 1. Relative rates by education level and place of residence for the oldest cohort are equal to 1.
